# Establishment of the first WHO International Standard for antiserum to Respiratory Syncytial Virus: Report of an international collaborative study

**DOI:** 10.1016/j.vaccine.2018.10.087

**Published:** 2018-11-29

**Authors:** Jacqueline U. McDonald, Peter Rigsby, Thomas Dougall, Othmar G. Engelhardt, Study Participants

**Affiliations:** a Division of Virology, National Institute for Biological Standards and Control (NIBSC), South Mimms, Potters Bar, Herts EN6 3QG, UK; b Biostatistics, National Institute for Biological Standards and Control (NIBSC), South Mimms, Potters Bar, Herts EN6 3QG, UK

**Keywords:** Respiratory Syncytial Virus, RSV, Neutralisation assay, International standard, Standardisation, Collaborative study

## Abstract

Respiratory Syncytial Virus (RSV), a leading cause of lower respiratory tract illness, has been a focus of vaccine development efforts in recent years. RSV neutralisation assays are particularly useful in the evaluation of immunogenicity of RSV vaccine candidates. Here we report a collaborative study that was conducted with the aim to establish the 1st International Standard for antiserum to RSV, to enable the standardisation of results across multiple assay formats. Two candidate standards were produced from serum samples donated by healthy adult individuals. 25 laboratories from 12 countries, including university laboratories, manufacturers/developers of RSV vaccines and public health laboratories, participated in the study. The study samples comprised the two candidate standards, NIBSC codes 16/284 and 16/322, naturally infected adult sera, age stratified naturally infected paediatric sera, sera from RSV vaccine clinical trials in maternal and elderly subjects, a monoclonal antibody to RSV (palivizumab), two cotton rat serum samples and samples from the BEI Resources panel of human antiserum and immune globulin to RSV.

The collaborative study showed that between-laboratory variability in neutralisation titres was substantially reduced when values were expressed relative to those of either of the two candidate international standards. Stability of 16/284 and 16/322 maintained for 6 months at different temperatures showed no significant loss of activity (relative to that at −20 °C storage temperature) at temperatures of up to +20 °C. Based on these results, 16/284 was established as the 1st International Standard for antiserum to RSV, with an assigned unitage of 1000 International Units (IU) of anti-RSV neutralising antibodies per vial, by the WHO Expert Committee on Biological Standardisation, with 16/322 suitable as a possible replacement standard for 16/284.

## 1 Introduction

Respiratory Syncytial Virus (RSV) is a leading cause of lower respiratory illness particularly in infants, the elderly and immunocompromised adults [1], [2], [3]. There is currently no vaccine to prevent RSV infection, but the development of a vaccine is recognised as a global priority by national governments, the World Health Organization (WHO), the pharmaceutical industry and not for profit health organizations.

Activity in this area has increased significantly in recent years, with at least 51 RSV vaccine candidates in development [4]. RSV neutralising activity in serum has been reported to correlate with protection against RSV acute lower respiratory infection in both rodent models and human infants [5]. Quantifying this neutralising activity is vital in the development of future RSV vaccines.

RSV neutralisation assays come in multiple formats and one of the challenges in RSV vaccine research is accurately comparing the neutralisation titres in sera from multiple clinical trials, each using a different neutralisation assay format [6]. A reference antiserum is needed to standardize clinical trials and outcomes. A multi-laboratory RSV neutralization assay survey study of 12 diverse assay formats was recently conducted and found that it was feasible to harmonize neutralization results using a standard [6]. Pooled human serum confirmed as seropositive for RSV was proposed as the candidate material to be assessed for its suitability to serve as a WHO International Standard (IS).

This study aimed to characterise two candidate anti-RSV sera in diverse RSV neutralisation assays to assess their suitability to be used as the 1st International Standard for anti-serum to RSV. Age stratified paediatric serum pools and vaccinee serum pools from 3 separate clinical trials were also evaluated to establish commutability of the standard. The BEI Resources panel of human antiserum and immune globulin to RSV (NR-32832) was also included to allow comparison with and potential calibration against the proposed international standard, as these materials are currently being used by some laboratories as internal standards.

## 2 Materials and methods

### 2.1 Preparation and testing of candidate standards

Serum samples donated by healthy adults were used as the source material to prepare the candidate international standards. The samples were classified as having low, medium or high RSV neutralising titre by a previous study [6]. Six samples with a high or medium neutralising titre, which were shown to improve agreement between laboratories when used individually to normalise results, were pooled to create each candidate standard. The serum pools were filtered, then filled in glass ampoules (0.5 mL per ampoule) and freeze dried to produce the candidate standards. Two candidate international standards were produced; NIBSC 16/284 and NIBSC 16/322.

The ampoules were sealed under high purity liquid nitrogen (99.99%) and stored at -20 °C. The mean oxygen headspace was measured non-invasively by frequency modulated spectroscopy and residual moisture content was measured using the colorimetric Karl Fischer method in a dry box environment (Table 1). Ampoules were tested at the beginning, middle and end of the freeze dry process in a neutralisation assay against RSV. There was no overall loss of neutralisation activity due to freeze drying.

**Table 1:** Product summary.

Production summary for candidate international standards		
NIBSC code	16/284	16/322
Mean fill mass	0.5239 g	0.5230 g
CV fill mass	0.95%	0.62%
Mean dry weight	0.0430 g	0.0443 g
CV of dry weight	0.27%	0.39%
Mean residual moisture	1.04%	0.32%
CV of residual moisture	25.50%	11.86%
Mean oxygen headspace	0.44%	0.44%
CV of oxygen headspace	26.02%	35.69%

### 2.2 Participants

26 laboratories were invited to participate in the study. 25 laboratories from twelve countries agreed to participate. 23 laboratories returned data; two of these returned 2 sets of data, one laboratory using two distinct assay methods and the other using two virus strains, giving a total of 25 datasets. Participants are listed in Table 2.

**Table 2:** List of collaborative study participants.

Pedro A Piedra Baylor College of Medicine Houston, TX, US	Koen Maleux/ Thi Lien Anh Nguyen GSK Vaccines Rixensart, Belgium	Marina Boukhvalova/ Kevin Yim Sigmovir Biosystems Rockville, MD, US
Edward Walsh University of Rochester Rochester, NY, US	Judy Beeler FDA Silver Spring, MD, US	Surender Khurana FDA Silver Spring, MD, US
Cindy Shambaugh MedImmune Mountain View, CA, US	Barney Graham NIAID, NIH Bethesda, MD, US	David Cooper Pfizer Pearl River, NY, US
Shabir Madhi NICD Johannesburg, South Africa	Carolin Schmittwolf Bavarian Nordic Munich, Germany	Carel van Baalen Viroclinics Rotterdam, The Netherlands
Stephen Glanville hVIVO London, UK	Elizabeth Stillman Aragen Biosciences Morgan Hill, CA, US	Othmar Engelhardt/ Jacqueline McDonald NIBSC Potters Bar, UK
Zi-Zheng Zheng/ Yong-Peng Sun Xiamen University Xiamen, China	Manki Song International Vaccine Institue Seoul, Korea	Myra Widjojoatmodjo Janssen Vaccines and Prevention Leiden, The Netherlands
Rik de Swart Erasmus University Medical Center Rotterdam, The Netherlands	Jose A. Melero Instituto de Salud Carlos III Madrid, Spain	Elly van Riet Intravacc Bilthoven, The Netherlands
Joyce Nyiro/ James Nokes Kemri-Wellcome Trust Research Programme Kilifi, Kenya	Rick Holtsberg Integrated Biotherapeutics Inc. Rockville, MD, US	

### 2.3 Assay methods

All participants used their own in-house assays and virus stocks for determining RSV neutralization antibody titres. The assays used in this study all use the same basic principle but vary on the details. Briefly, virus and sample are mixed together, this mixture is then added to cells and the level of viral infectivity is measured; the lower the viral infectivity, the greater the neutralisation titre of the sample. A summary of the different assays used in this study is shown in supplementary Table 1.

### 2.4 Sample panel

A total of 38 samples were available to participants. The samples were shipped in dry-ice and storage at ≤−20 °C was recommended. All participating laboratories were provided with the core panel and the panel for commutability listed in Table 3, except for 2 laboratories that were unable to receive the cotton rat and paediatric samples due to internal processes. Of the 25 laboratories, 19 were able to receive the BEI Resources panel. Six laboratories were not able to receive this panel due to administrative reasons.

**Table 3:** Samples Included in the Collaborative Study.

Sample panels	Name	Sample code
Core human panel	International Standard Candidate 1- NIBSC 16/284	11
	International Standard Candidate 2 – NIBSC 16/322	24
	International Standard Candidate 1 Pre-lyophilised	9
	International Standard Candidate 1 Pre-lyophilised	29
	International Standard Candidate 2 Pre-lyophilised	18
	P-0015 Individual Adult Human Serum	1
	P-0015 Individual Adult Human Serum	3
	P-0029 Individual Adult Human Serum	31
	P-0035 Individual Adult Human Serum	38
	P-0041 Individual Adult Human Serum	37
	P-0056 Individual Adult Human Serum	16
	P-0056 Individual Adult Human Serum	26

Core monoclonal antibody panel	Palivizumab 0.1 mg/ml	10
	Palivizumab 1 mg/ml	22

Core animal panel	Cotton Rat Serum Pool - RSV A	27
	Cotton Rat Serum Pool - RSV B	13

Panel for commutability – vaccinee	Maternal sera pool from RSV F Trial – 1	6
	Maternal sera pool from RSV F Trial – 2	20
	Maternal sera pool from RSV F Trial – 3	17
	Maternal sera pool from RSV F Trial – 4	15
	Maternal sera pool from RSV F Trial – 5	4
	Elderly sera pool from RSV F Trial – 1	21
	Elderly sera pool from RSV F Trial – 2	25
	Elderly sera pool from RSV F Trial – 3	12
	Elderly sera pool from RSV F Trial – 4	34
	Adult sera pool from RSV F Trial – 1 (Low)	28
	Adult sera pool from RSV F Trial – 2 (Med)	35
	Adult sera pool from RSV F Trial – 3 (High)	30

Panel for commutability – paediatric	Paediatric Serum Pool Age <1 year	5
	Paediatric Serum Pool Age 1–2 years	23
	Paediatric Serum Pool Age 2–3 years	33
	Paediatric Serum Pool Age 3–4 years	8

BEI resources panel	NR-4020: Human Reference Antiserum to RSV	7
	NR-4021: Human Antiserum to RSV, High Control	19
	NR-4022: Human Antiserum to RSV, Medium Control	2
	NR-4023: Human Antiserum to RSV, Low Control	36
	NR-21973: Human Reference Immunoglobulin to RSV	14
	NR-49447: Human IgG-Depleted Serum (Negative control)	32

### 2.5 Study plan

Participants were requested to:

– Follow the recommended protocols for storage and reconstitution of the study samples.
– Determine the neutralisation titre against RSV of each of the samples in the panel by performing four independent assays using their in-house method and using in-house reagents, including their own virus stocks.
– Avoid multiple freeze-thaw cycles of the study samples.

Participants were requested to report their results electronically using standard forms provided by the study coordinator. The forms requested both raw data and calculated endpoint titres.

### 2.6 Statistical analysis

Analysis was performed using ED50s reported by the participants or calculated at NIBSC and also using relative potencies, i.e. ED50s expressed relative to candidate standard samples. All ED50s and relative potency estimates were combined as Geometric Means (GM) and variability within laboratories (between assays) and between laboratories was expressed using Geometric Coefficients of Variation (%GCV), i.e. (10 ^s^-1) × 100%, where s is the standard deviation of the log_10_ ED50s or potency estimates. In some cases, the endpoint ED50 was not covered by the range of dilutions used by the participant and results were reported as “less than” or “greater than” and all estimates for these samples in that laboratory were excluded from further analysis. Any exclusions due to high intra-assay or inter-assay variability within a laboratory are described in the results section of this report.

An exploratory visual assessment of sample and laboratory differences was carried out by performing a simple correspondence analysis of potencies relative to candidate standard sample 11 (16/284). Further assessment of agreement in geometric mean potencies for each pair of laboratories was performed by calculating Lin’s concordance correlation coefficient with log transformed potencies relative to candidate standard samples 11 or 24 (16/284 and 16/322 respectively). These were calculated using the R package ‘DescTools’ [7].

Relative potency estimates obtained for accelerated thermal degradation samples were used to fit Arrhenius equations relating the degradation rate to absolute temperature assuming first-order decay and hence predict the degradation rates when stored at −20 °C [8].

### 2.7 Laboratories 7, 11a & 13

These laboratories reported their data in a format other than ED50 or equivalent. To allow like for like comparisons, ED50 values for these datasets were calculated using the dose-response assay data supplied by the laboratories. The re-calculation could affect the within-assay variability for these laboratories, as the assays were not optimised to calculate an ED50.

## 3 Results

### 3.1 Data returned

A total of 25 datasets were received from 23 participants. Each laboratory was allocated a randomly assigned numerical code (n = 23), not representing the order of the list in Table 2. Where laboratories returned 2 datasets, the datasets were designated a or b. Laboratory 11 returned 2 datasets using 2 different neutralisation assay formats, and laboratory 14 returned 2 datasets using 2 different virus strains. The data from laboratory 6 were not included in the analysis, as the results returned did not give estimated values for either candidate standard but instead returned values of >1024. All laboratories tested their sample panel in 4 individual assays.

### 3.2 Intra-assay and inter-assay variability in ED50s

**Fig. 1:**
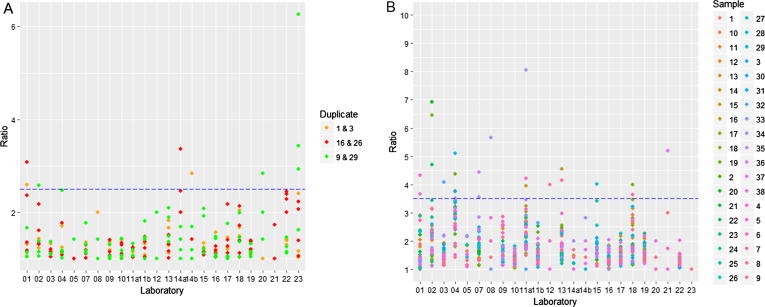
**Intra-assay and inter-assay variability**. Ratios of ED50s for coded duplicates in each assay and the cut-off (blue dash line) for acceptability (A). Ratios of the maximum and minimum ED50s for each sample in each laboratory and the cut-off for acceptability (blue dash line) (B).

Intra-assay variability was assessed using the coded duplicate samples included in the study. Three pairs of coded duplicates were included in the panel of samples, these were; 1 & 3, 9 & 29, and 16 & 26. Where any of the coded duplicate ED50s within an assay differed by more than a factor of 2.5, no result for that assay was used (Fig. 1a), which was the case for ∼8% of assays.

Inter-assay variability was assessed for each sample from the ratio of the maximum and minimum ED50 results across all assays within a laboratory. Where the ratio for a sample exceeded 3.5, all results for that sample for that laboratory were excluded (Fig. 1b). Further to the excluded assays described above, this accounted for ∼5% of cases.

### 3.3 Inter-laboratory variability in ED50s and estimates of relative potencies

**Fig. 2:**
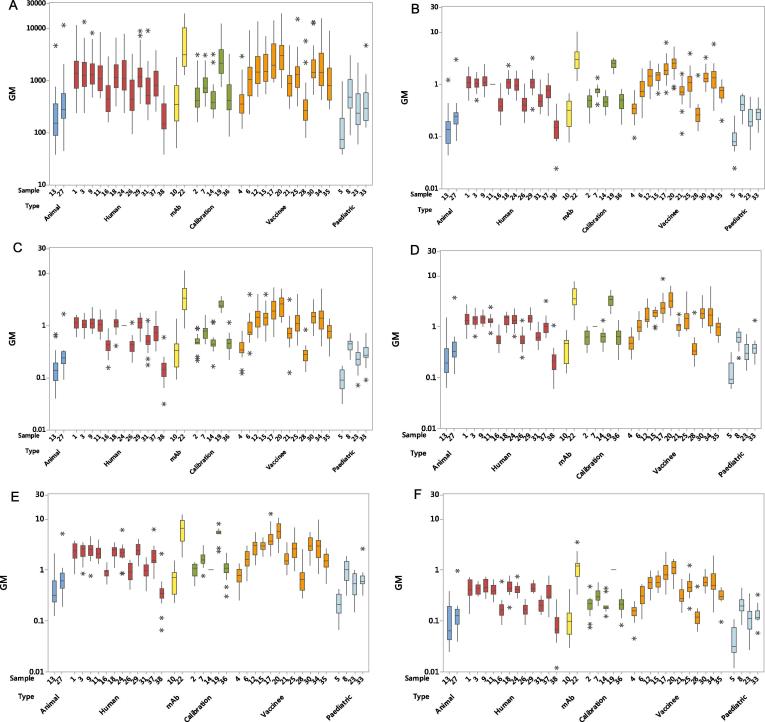
**Inter-laboratory variability in ED50s and estimates of relative potencies.** Laboratory geometric mean ED50s (A), potencies relative to 16/284 (B), potencies relative to 16/322 (C), potencies relative to sample 7 (D), potencies relative to sample 14 (E) and potencies relative to sample 19 (F). Sample groupings are represented as different colour boxes (dark blue = animal; red = human; yellow = monoclonal antibodies; green = BEI samples for calibration; orange = vaccine; pale blue = paediatric). *indicates outliers.

Variability between laboratories for ED50s and potencies relative to different candidate standards was assessed using the inter-laboratory GCV values and ratios of the maximum and minimum estimates calculated (Table 4, Fig. 2, Supplementary Table 2). A summary of the calculated GCVs can be found in Table 5. An IgG deficient serum sample, included as a negative control (sample 32), will be excluded from all further discussions of data unless specifically related to this sample. The data was split into 2 sets based on the concordance correlation coefficients described below. Briefly, set A includes all the data and set B excludes laboratories showing poor concordance, which was defined as those having concordance correlation values of <0.8 with more than 50% of all other labs.

**Table 4:** Sample geometric mean ED50 and potency estimates relative to 16/284 or 16/322.

Type	Sample	ED50				Potencies v 16/284				Potencies v 16/322			
		GM	Max:Min	GCV	N	GM	Max:Min	GCV	N	GM	Max:Min	GCV	N
Animal	13	217	123	240	21	0.15	28	131	21	0.15	17	107	21
	27	396	257	227	20	0.26	36	110	20	0.25	18	76	20

Human	1	1440	47	155	24	1.05	4	42	24	1.06	4	41	24
	3	1418	56	156	24	1.03	3	39	24	1.04	3	31	24
	9	1581	17	114	23	1.11	4	40	23	1.09	3	33	23
	11	1372	18	125	24					1.01	4	43	24
	16	556	26	158	24	0.41	6	52	24	0.41	6	53	24
	18	1369	20	126	23	1.05	4	38	23	1.04	5	42	23
	24	1364	32	157	24	0.99	4	43	24				
	26	575	34	177	24	0.42	6	52	24	0.42	6	44	24
	29	1407	25	143	23	1.08	10	54	23	1.06	4	37	23
	31	713	51	148	23	0.50	4	48	23	0.51	7	55	23
	37	1067	20	135	22	0.79	13	68	22	0.76	6	58	22
	38	220	44	166	23	0.16	45	108	23	0.15	22	89	23

mAb	10	362	55	164	24	0.26	8	90	24	0.27	17	103	24
	22	3882	15	129	21	2.92	9	81	21	2.94	13	97	21

BEI Resources panel	2	545	20	138	19	0.47	5	48	19	0.45	5	54	19
	7	901	12	98	19	0.78	3	27	19	0.75	4	37	19
	14	543	17	136	18	0.47	3	31	18	0.46	7	55	18
	19	2748	16	116	14	2.48	3	33	14	2.37	3	29	14
	32	8	104	495	5	0.01	11	164	5	0.01	7	117	5
	36	579	37	172	18	0.49	6	63	18	0.48	5	47	18

Paediatric	5	120	25	178	20	0.09	10	68	20	0.09	5	63	20
	8	631	33	159	20	0.42	5	59	20	0.43	3	37	20
	23	311	38	182	22	0.22	10	75	22	0.23	7	56	22
	33	392	37	169	22	0.28	11	63	22	0.29	9	64	22

Vaccinee	4	421	24	129	22	0.34	8	59	22	0.35	7	55	22
	6	1115	40	179	21	0.80	9	71	21	0.84	14	70	21
	12	1806	27	142	22	1.34	5	53	22	1.32	7	62	22
	15	1849	13	111	22	1.37	4	39	22	1.37	8	57	22
	17	2715	15	129	23	1.92	9	63	23	1.87	9	69	23
	20	3219	27	127	21	2.42	7	67	21	2.43	4	57	21
	21	986	16	125	24	0.72	14	71	24	0.72	26	76	24
	25	1433	47	138	23	1.10	11	72	23	1.08	10	68	23
	28	368	72	186	21	0.28	12	72	21	0.27	6	52	21
	30	1905	24	139	22	1.41	5	47	22	1.39	5	45	22
	34	1806	36	165	20	1.33	19	94	20	1.32	12	92	20
	35	975	31	139	24	0.71	7	52	24	0.71	5	53	24

GM: Geometric Mean.

Max:Min: Ratio of maximum and minimum laboratory geometric means.

GCV: Geometric Coefficient of Variation (%).

N: Number of laboratories used in calculation of GM and GCV.

**Table 5:** Summary of inter-laboratory GCV values (%); shading indicates level of inter-laboratory variability.

Shading indicates level of inter-laboratory variability: darker red = increased variability.

Blue shading: animal and mAb samples are shaded in blue as they behave differently to human serum samples.

There was a large variation in estimated geometric mean titres reported from laboratories, with GCVs ranging from 98% to 240% (set A) or 54% to 173% (set B) (Table 5, Fig. 2a). When the potencies were expressed relative to either of the candidate standards or to samples 7, 14 or 19, much greater agreement was seen between the different laboratories (Table 5, Fig. 2b–f). Expressed relative to 16/284, the GCVs ranged from 27% to 131% (set A) or 15% to 83% (set B); expressed relative to 16/322, the GCVs ranged from 29% to 107% (set A) or 16% to 95% (set B); expressed relative to sample 7, the GCVs ranged from 17% to 142% (set A) or 7% to 121% (set B); expressed relative to sample 14, the GCVs ranged from 22% to 125% (set A) or 10% to 95% (set B); expressed relative to sample 19, the GCVs ranged from 22% to 153% (set A) or 10% to 98% (set B) (Table 5).

### 3.4 Concordance correlation coefficient

**Fig. 3:**
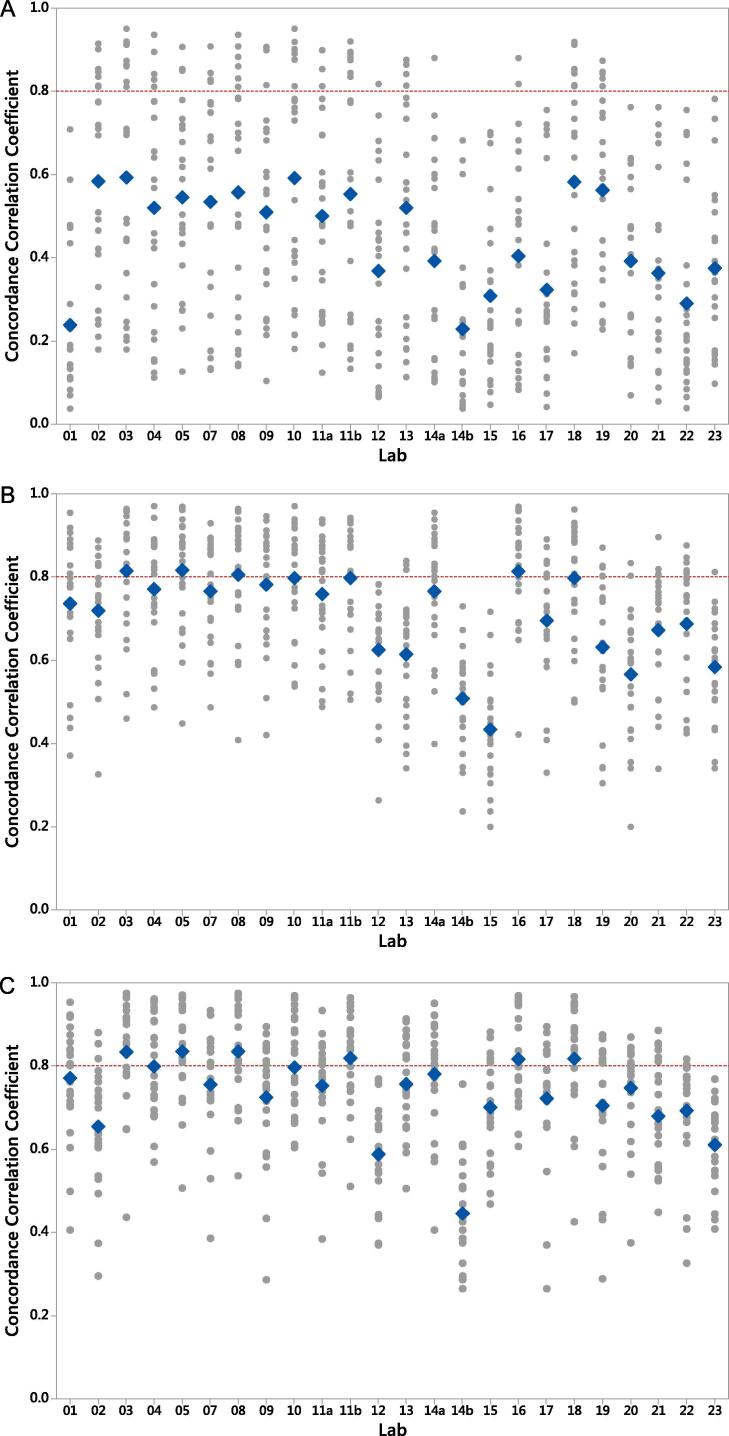
**Concordance Correlation Coefficients.** Concordance correlation coefficients for the human samples only (A), log potencies relative to 16/284 (B), and log potencies relative to 16/322 (C). Red line indicates threshold of “poor” concordance correlation coefficients <0.8. Blue diamond indicates mean concordance correlation coefficient.

To assess the level of agreement between all pairs of laboratories, concordance correlation coefficients were calculated using the log titres or log potencies for the samples in the human panel, including the candidate standards (Fig. 3, Supplementary Tables 3 and 4). For the purpose of assessing the level of agreement that may be achieved between several laboratories, concordance of >0.8 was considered “good” and a laboratory was designated as having “poor” concordance when concordance correlation coefficients were <0.8 with more than 50% of the other laboratories. When potencies were expressed as absolute ED50s, all laboratories showed poor concordance values (Fig. 3a). However, concordance values for all laboratories improved when expressed as relative to either of the candidate standards, but concordance was noted to remain poor for laboratories 02, 12, 13, 14b, 15, 17, 19, 20, 21, 22 and 23 (Fig. 3b, c). When looking at inter-laboratory variability, it was found that removing these 11 laboratories further reduced variability in the data (Table 5, Set B).

### 3.5 Correspondence analysis

**Fig. 4:**
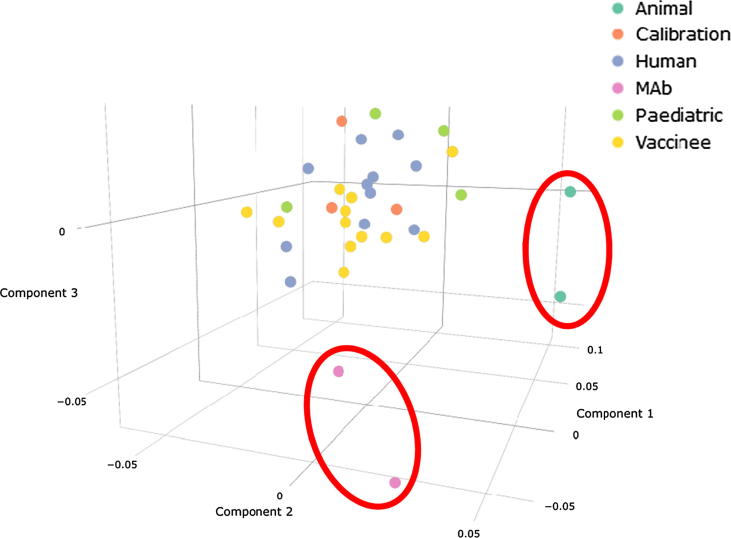
**Correspondance analysis scatterplot.** Scatterplot of row (sample) principle coordinates from correspondence analysis. Samples exhibiting similar patterns in results across different laboratories will lie in close proximity on the plot.

A simple correspondence analysis of log transformed potencies relative to 16/284 was carried out after exclusion of sample 32 (negative), samples 14, 19 and labs 07, 12, 15, 21 due to lack of available data (≤80% of labs or samples with a reported result). This multivariate statistical technique allows an exploratory visual assessment of the results profiles observed for samples (across different laboratories) or laboratories (across different samples). A scatterplot of the first three sample components is shown in Fig. 4. Samples exhibiting similar results profiles across different laboratories would be expected to be in close proximity on this plot. In this case, there is evidence that results profiles across laboratories are different for the animal and monoclonal antibody (mAb) panel samples. There is no data based explanation for this difference and further work with a greater number of samples in each grouping is required to assess this difference. No particular groupings of laboratories were observed and plots of laboratory components are not shown.

### 3.6 Stability studies

After 6 months storage at elevated temperatures, two independent assays were performed for each temperature, and potencies relative to the −20 °C baseline for 16/284 and 16/322 were obtained (Supplementary Tables 6 and 7). These accelerated degradation studies were used to predict the rate at which the samples lose neutralisation activity (Table 6). The rates could not be calculated for 16/322 as the accelerated degradation samples had not lost sufficient activity in the initial 6 month period. For 16/284, the data show that there is no loss of activity at +4 °C and minimal loss at +20 °C (temperatures used during laboratory manipulation for assays), relative to the −20 °C baseline. The low predicted loss in activity per year (<0.01%) when stored at −20 °C suggests 16/284 is sufficiently stable to serve as a WHO International Standard. The stability of 16/284 and 16/322 will be monitored regularly throughout the lifetime of the standards.

**Table 6:** Thermal degradation assessment of 16/284; estimated percentage loss per month and year.

Storage temperature (°C)	% loss per month	% loss per year
−20	0.001	0.009
4	0.047	0.561
20	0.516	6.016
37	4.923	45.435

The stability of candidate standards reconstituted in liquid form was also assessed. Ampoules of the three candidate materials that had been reconstituted in 0.5 mL of sterile glass distilled water and maintained at different temperatures were tested. Two independent assays were performed at each temperature and time point, and potencies relative to that of an ampoule stored at −20 °C and freshly reconstituted were obtained for both candidates. The data suggest that there is no loss of activity for any of the reconstituted materials that had been stored at +4 °C or +22 °C for both standards for up to a week. After 4 weeks at +4 °C, 16/284 had lost 24% of its neutralisation activity. At +37 °C, 16/284 showed approximately 20% loss after 2 weeks, and 16/322 had lost >40% activity after 1 week (Supplementary Tables 8 and 9).

## 4 Discussion

As of November 2017, there are 18 RSV vaccine candidates in human clinical trials, with 5 in phase 2 and 1 in phase 3 trials [4]. It is reasonable to suggest that a licensed RSV vaccine is not that far away and, therefore, a reliable way to consistently test the immunogenicity of any vaccine on or entering the market is required. Neutralisation assays are routinely used as a test of immunogenicity and an International Standard to allow comparison of results from different assays was not available. In this study we describe the production of 2 such candidates for an International Standard and the results from a collaborative study used to test their suitability.

The data from this study show that both 16/284 and 16/322 are suitable as reference standards to measure RSV neutralisation activity in a range of sample types, particularly human serum, due to their ability to reduce variability across the various assay methods included in this study. We proposed 16/284 as the first International Standard (IS) for antiserum to RSV, with an assigned potency of 1000 IU/ampoule. We also proposed the use of 16/322 as a possible replacement standard, once 16/284 has been depleted.

The data generated showed high levels of variability across the laboratories for all sample types. This can be attributed to the varying formats and methods used to assess RSV neutralizing antibody titres. A deliberate decision was made that no aspect of the methods used would be controlled, to mimic the real world variation of the assays currently being used. However, once the data were expressed as relative potencies (relative to the candidate standards), the variability between laboratories decreased. Variability was decreased further with the exclusion of additional laboratories that showed poor concordance. However, there was no common aspect in the methods between the excluded laboratories, or an obvious reason why concordance correlation coefficients should be low for these laboratories, and to maintain an accurate picture of the variability of RSV neutralisation assays, all data (set A) were used for calculations relating to the International Standard. The two exceptions to the above were laboratory 14b, which looked at neutralisation titres against RSV B, and laboratory 17 which included guinea pig complement in their assay. Further collaborative studies will be needed to determine the usefulness of this standard against RSV B viruses and assays using animal complement. Set B suggests that further improvement in agreement between laboratories may be possible with standardisation of the assay or assay materials.

Stability data for 16/284, based on storage at elevated temperatures for up to six months, gave a low predicted loss in activity per year (<0.01%) when stored at -20 °C, suggesting suitable stability to serve as a WHO IS. A long term programme of monitoring stability will be needed to show that 16/284 remains stable over its lifetime. Stability data for 16/322 indicated that the candidate is stable over a 6 month period and there was insufficient loss of activity to determine a rate of loss. Furthermore, stability analysis showed that the candidate standards were also stable after reconstitution, but for the best results it is recommended that the standards are used on the same day as reconstitution.

The BEI Resources panel consisted of a panel of human antisera and immunoglobulin. These were included to assess their ability to act as working standards and data from the collaborative study support their suitability, as they are able to reduce inter-laboratory variability when used as standards.

Commutability for vaccine serum samples and paediatric samples was assessed in this collaborative study. Sera from both maternal and elderly clinical trials were included and both candidate standards were able to reduce GCVs for these samples. Pools of sera from naturally infected paediatric subjects were also assessed and both candidates were able to reduce the GCVs across all laboratories for these samples. Vaccinee sera from paediatric clinical trials were not available for testing but it is reasonable to suggest that as the standards were suitable for sera from naturally infected children, they will be suitable for testing paediatric vaccine sera.

As well as the inter-laboratory GCVs, correspondence analysis did not suggest differences in the behaviours of the vaccinee sera and paediatric sera in comparison to the sera from naturally infected adults, which further indicates that the candidate standards are commutable with these sample types.

This study achieved its stated aims and an International Standard was proposed, with an International Unitage (IU). The standard is useful for multiple sample types across a wide variety of assay formats; however, the analysis suggests that the cotton rat serum samples and monoclonal antibody samples behave differently from the human serum samples, and that a more suitable standard may need to be considered for those sample types.

## 5 Conclusions

Based on the results of this study, the serum preparation 16/284 was deemed suitable to serve as an International Standard for the testing of human serum samples in RSV neutralisation assays. As such this preparation was established by the WHO Expert Committee on Biological Standardisation (ECBS) as the first WHO International Standard for antiserum to RSV, with an assigned potency of 1000 IU per ampoule.
