# Supplementary material for: Establishment of the first WHO International Standard for antiserum to Respiratory Syncytial Virus: Report of an international collaborative study
Source: Vaccine. 2018 Nov 29;36(50):7641–9. doi: 10.1016/j.vaccine.2018.10.087 (PMC6838659; doi:10.1016/j.vaccine.2018.10.087)
Supplement: Supplementary data 2 [file mmc2.docx]

**Supplementary Tables**

**Table S1**. Details of methods used by participants

| **Lab Code** | **Duration of Assay** | **Cell Line** | **Complement/ % and type** | **RSV Strain** | **Conc. / Amount Virus added** | **Read-out** |
| --- | --- | --- | --- | --- | --- | --- |
| **01** | 24 - 48 hours | Vero | No | A2 Recombinant GFP | 5,000 pfu/cell | GFP-fluorescence |
| **02** | 2 days | HEp-2 | No | A2 | 200 pfu/well | Plaque counts |
| **03** | 2 days | A549 | No | Recombinant FFL-A2 | 2x10^6^ PFU/ml | Firefly luciferase |
| **04** | 3 days | Vero | No | A2 | 1,000 pfu | ELISA A450 |
| **05** | 3 days | HEp-2 | No | A2 | 75 pfu | F protein EIA |
| **06** | 24 hours | HEp-2 | No | A2 | 100 pfu/well | Plaque counts |
| **07** | 6 days | Vero | No | RSV A (long) | 100 pfu/well | Fluorescent plaque count |
| **08** | 6-7 days | HEp-2 | No | RSV/A/Tracy | 3^3^ TCID100/.05 or 3^5.5^ TCID50 | CPE |
| **09** | 4 days | Vero | No | rRSV/V35pX EGFP | 3,750 pfu/mL | Plaque counts by spots with fluorescence |
| **10** | 24 hours | Vero | No | A2 Recombinat GFP | 652 ffu/well | Foci/well |
| **11a** | 5 days | HEp-2 | No | A2 | 25-50 pfu | Plaque counts |
| **11b** | 4 days | Vero-81 | No | A2 | 500-1,000 PFU/well | OD at 450nm |
| **12** | 7 days | Vero | No | A2 | 200 pfu/well | Plaque counts |
| **13** | 24 hours | Vero | No | A2 | 500-5,000 TCID50/well | RSV N gene amplicon |
| **14a** | 6 days | HEp-2 | No | RSV/A (Long) | 100 TCID50 | CPE |
| **14b** | 6 days | HEp-2 | No | RSV/B (B1 WT) | 100 TCID50 | CPE |
| **15** | 3 days | A549 | No | RSV A (M37) | 550 ffu/well | Fluorescent foci |
| **16** | 7 days | Vero E6 | No | A2 | 70 – 130 pfu/well | Plaque counts |
| **17** | 2 days | A549 | 5% guinea pig complement | A2 expressing Renilla Luciferase | 250 pfu/100 ul/well | Luciferase activity |
| **18** | 24 hours | HEp-2 | No | A2 expressing mKate | MOI=4, 5×10^4^ cells/well | Mean fluorescence intensity per well |
| **19** | 24 hours | HEp-2 | No | Recombinant mKate-RSV (A2) | 2x10^4^ pfu/well | Fluorescence Intensity |
| **20** | 7 days | HEp-2 | No | RSV A (long) | 3500 pfu/ml | CPE |
| **21** | 5 days | HEp-2 | No | RSV-A Memphis 37b | ~2.75 log10 TCID50/ml | CPE |
| **22** | 6-8 days | HEp-2 | No | A2 | 50 pfu/well | Plaque counts |
| **23** | 4-5 days | HEp-2 | No | A2 | 150 pfu/well | Plaque counts |

**Table S2.** Sample geometric mean potency estimates relative to BEI materials samples 7, 14 and 19

| Type | Sample | Potencies v Sample 7 | | | | Potencies v Sample 14 | | | | Potencies v Sample 19 | | | |
| --- | --- | --- | --- | --- | --- | --- | --- | --- | --- | --- | --- | --- | --- |
|  |  | GM | Max:Min | GCV | N | GM | Max:Min | GCV | N | GM | Max:Min | GCV | N |
| Animal | 13 | 0.26 | 24 | 141 | 12 | 0.40 | 17 | 124 | 12 | 0.08 | 16 | 153 | 12 |
|  | 27 | 0.41 | 32 | 142 | 11 | 0.66 | 22 | 125 | 11 | 0.12 | 24 | 126 | 11 |
| Human | 1 | 1.37 | 5 | 57 | 13 | 2.14 | 4 | 55 | 13 | 0.42 | 5 | 55 | 13 |
|  | 3 | 1.37 | 4 | 39 | 13 | 2.15 | 4 | 49 | 13 | 0.42 | 2 | 26 | 13 |
|  | 9 | 1.52 | 2 | 24 | 13 | 2.37 | 3 | 33 | 13 | 0.47 | 3 | 38 | 13 |
|  | 11 | 1.32 | 2 | 25 | 13 | 2.06 | 2 | 25 | 13 | 0.41 | 3 | 34 | 13 |
|  | 16 | 0.55 | 3 | 34 | 13 | 0.86 | 3 | 30 | 13 | 0.17 | 7 | 63 | 13 |
|  | 18 | 1.42 | 3 | 33 | 13 | 2.22 | 3 | 38 | 13 | 0.44 | 4 | 44 | 13 |
|  | 24 | 1.36 | 4 | 38 | 13 | 2.12 | 4 | 41 | 13 | 0.42 | 3 | 30 | 13 |
|  | 26 | 0.53 | 4 | 45 | 13 | 0.83 | 4 | 48 | 13 | 0.16 | 3 | 40 | 13 |
|  | 29 | 1.45 | 3 | 31 | 13 | 2.27 | 3 | 41 | 13 | 0.45 | 2 | 22 | 13 |
|  | 31 | 0.64 | 3 | 28 | 13 | 0.99 | 3 | 37 | 13 | 0.20 | 2 | 35 | 13 |
|  | 37 | 1.08 | 5 | 54 | 13 | 1.69 | 9 | 72 | 13 | 0.33 | 6 | 63 | 13 |
|  | 38 | 0.23 | 18 | 96 | 13 | 0.36 | 32 | 112 | 13 | 0.07 | 22 | 116 | 13 |
| mAb | 10 | 0.32 | 7 | 91 | 13 | 0.51 | 5 | 80 | 13 | 0.10 | 14 | 98 | 13 |
|  | 22 | 3.55 | 6 | 82 | 13 | 5.55 | 7 | 79 | 13 | 1.10 | 11 | 85 | 13 |
| Calibration | 2 | 0.63 | 3 | 39 | 13 | 0.99 | 3 | 39 | 13 | 0.20 | 6 | 65 | 13 |
|  | 7 |  |  |  |  | 1.56 | 2 | 22 | 13 | 0.31 | 3 | 36 | 13 |
|  | 14 | 0.64 | 2 | 22 | 13 |  |  |  |  | 0.20 | 4 | 41 | 13 |
|  | 19 | 3.23 | 3 | 36 | 13 | 5.05 | 4 | 41 | 13 |  |  |  |  |
|  | 32 | 0.01 | 9 | 150 | 4 | 0.01 | 7 | 131 | 4 | 0.00 | 20 | 259 | 4 |
|  | 36 | 0.64 | 5 | 55 | 12 | 1.00 | 7 | 72 | 12 | 0.20 | 5 | 55 | 12 |
| Paediatric | 5 | 0.13 | 5 | 77 | 11 | 0.20 | 6 | 81 | 11 | 0.04 | 9 | 94 | 11 |
|  | 8 | 0.60 | 4 | 50 | 11 | 0.95 | 5 | 66 | 11 | 0.19 | 5 | 57 | 11 |
|  | 23 | 0.31 | 5 | 79 | 12 | 0.49 | 6 | 87 | 12 | 0.10 | 13 | 94 | 12 |
|  | 33 | 0.41 | 5 | 54 | 12 | 0.65 | 8 | 71 | 12 | 0.13 | 6 | 54 | 12 |
| Vaccinee | 4 | 0.48 | 4 | 46 | 13 | 0.75 | 5 | 64 | 13 | 0.15 | 5 | 55 | 13 |
|  | 6 | 0.98 | 3 | 44 | 13 | 1.53 | 5 | 57 | 13 | 0.30 | 5 | 59 | 13 |
|  | 12 | 1.79 | 4 | 48 | 13 | 2.80 | 4 | 56 | 13 | 0.55 | 4 | 49 | 13 |
|  | 15 | 1.86 | 2 | 17 | 12 | 2.91 | 2 | 28 | 12 | 0.58 | 3 | 36 | 12 |
|  | 17 | 2.80 | 6 | 64 | 13 | 4.37 | 7 | 73 | 13 | 0.87 | 7 | 64 | 13 |
|  | 20 | 3.42 | 4 | 51 | 13 | 5.34 | 5 | 63 | 13 | 1.06 | 4 | 49 | 13 |
|  | 21 | 0.97 | 2 | 28 | 13 | 1.52 | 3 | 41 | 13 | 0.30 | 5 | 49 | 13 |
|  | 25 | 1.55 | 6 | 63 | 13 | 2.42 | 7 | 74 | 13 | 0.48 | 7 | 63 | 13 |
|  | 28 | 0.40 | 9 | 73 | 13 | 0.62 | 9 | 80 | 13 | 0.12 | 8 | 66 | 13 |
|  | 30 | 1.96 | 4 | 44 | 13 | 3.07 | 5 | 55 | 13 | 0.61 | 3 | 35 | 13 |
|  | 34 | 1.82 | 8 | 94 | 11 | 2.80 | 12 | 119 | 11 | 0.56 | 13 | 92 | 11 |
|  | 35 | 0.95 | 3 | 40 | 13 | 1.48 | 5 | 58 | 13 | 0.29 | 5 | 47 | 13 |

**Table S3.** Concordance correlation coefficients for log titres (human panel samples only); values ≥0.8 shaded

| **Lab** | **01** | **02** | **03** | **04** | **05** | **07** | **08** | **09** | **10** | **11a** | **11b** | **12** | **13** | **14a** | **14b** | **15** | **16** | **17** | **18** | **19** | **20** | **21** | **22** | **23** |
| --- | --- | --- | --- | --- | --- | --- | --- | --- | --- | --- | --- | --- | --- | --- | --- | --- | --- | --- | --- | --- | --- | --- | --- | --- |
| **01** |  |  |  |  |  |  |  |  |  |  |  |  |  |  |  |  |  |  |  |  |  |  |  |  |
| **02** | 0.18 |  |  |  |  |  |  |  |  |  |  |  |  |  |  |  |  |  |  |  |  |  |  |  |
| **03** | 0.18 | 0.92 |  |  |  |  |  |  |  |  |  |  |  |  |  |  |  |  |  |  |  |  |  |  |
| **04** | 0.11 | 0.77 | 0.81 |  |  |  |  |  |  |  |  |  |  |  |  |  |  |  |  |  |  |  |  |  |
| **05** | 0.29 | 0.72 | 0.71 | 0.46 |  |  |  |  |  |  |  |  |  |  |  |  |  |  |  |  |  |  |  |  |
| **07** | -0.06 | -0.34 | -0.42 | -0.51 | -0.18 |  |  |  |  |  |  |  |  |  |  |  |  |  |  |  |  |  |  |  |
| **08** | 0.13 | 0.77 | 0.82 | 0.83 | 0.48 | -0.45 |  |  |  |  |  |  |  |  |  |  |  |  |  |  |  |  |  |  |
| **09** | 0.14 | 0.81 | 0.86 | 0.94 | 0.50 | -0.51 | 0.91 |  |  |  |  |  |  |  |  |  |  |  |  |  |  |  |  |  |
| **10** | 0.23 | 0.71 | 0.70 | 0.42 | 0.91 | -0.24 | 0.47 | 0.48 |  |  |  |  |  |  |  |  |  |  |  |  |  |  |  |  |
| **11a** | 0.18 | 0.90 | 0.95 | 0.78 | 0.78 | -0.40 | 0.75 | 0.78 | 0.73 |  |  |  |  |  |  |  |  |  |  |  |  |  |  |  |
| **11b** | 0.19 | 0.69 | 0.70 | 0.44 | 0.85 | -0.21 | 0.54 | 0.48 | 0.90 | 0.76 |  |  |  |  |  |  |  |  |  |  |  |  |  |  |
| **12** | 0.13 | 0.83 | 0.92 | 0.90 | 0.59 | -0.34 | 0.84 | 0.88 | 0.55 | 0.89 | 0.61 |  |  |  |  |  |  |  |  |  |  |  |  |  |
| **13** | 0.07 | 0.42 | 0.45 | 0.59 | 0.23 | -0.29 | 0.68 | 0.66 | 0.21 | 0.40 | 0.25 | 0.48 |  |  |  |  |  |  |  |  |  |  |  |  |
| **14a** | 0.11 | 0.81 | 0.86 | 0.84 | 0.54 | -0.51 | 0.77 | 0.78 | 0.56 | 0.88 | 0.58 | 0.88 | 0.46 |  |  |  |  |  |  |  |  |  |  |  |
| **14b** | 0.11 | 0.47 | 0.48 | 0.57 | 0.27 | -0.31 | 0.64 | 0.69 | 0.25 | 0.42 | 0.26 | 0.48 | 0.74 | 0.42 |  |  |  |  |  |  |  |  |  |  |
| **15** | 0.04 | 0.25 | 0.21 | 0.34 | 0.13 | -0.18 | 0.33 | 0.38 | 0.10 | 0.21 | 0.12 | 0.24 | 0.63 | 0.24 | 0.60 |  |  |  |  |  |  |  |  |  |
| **16** | 0.43 | 0.24 | 0.23 | 0.15 | 0.47 | -0.08 | 0.18 | 0.17 | 0.37 | 0.27 | 0.35 | 0.19 | 0.08 | 0.18 | 0.10 | 0.05 |  |  |  |  |  |  |  |  |
| **17** | 0.08 | 0.49 | 0.49 | 0.66 | 0.27 | -0.30 | 0.61 | 0.72 | 0.25 | 0.44 | 0.24 | 0.51 | 0.82 | 0.48 | 0.88 | 0.68 | 0.09 |  |  |  |  |  |  |  |
| **18** | 0.71 | 0.27 | 0.25 | 0.16 | 0.43 | -0.07 | 0.16 | 0.18 | 0.36 | 0.26 | 0.27 | 0.18 | 0.07 | 0.18 | 0.11 | 0.04 | 0.69 | 0.11 |  |  |  |  |  |  |
| **19** | 0.24 | 0.85 | 0.91 | 0.64 | 0.85 | -0.35 | 0.69 | 0.70 | 0.82 | 0.92 | 0.81 | 0.77 | 0.34 | 0.73 | 0.41 | 0.17 | 0.32 | 0.38 | 0.31 |  |  |  |  |  |
| **20** | 0.24 | 0.85 | 0.87 | 0.74 | 0.68 | -0.33 | 0.75 | 0.83 | 0.61 | 0.81 | 0.57 | 0.78 | 0.44 | 0.65 | 0.59 | 0.23 | 0.25 | 0.54 | 0.29 | 0.84 |  |  |  |  |
| **21** | 0.47 | 0.41 | 0.36 | 0.22 | 0.64 | -0.12 | 0.26 | 0.26 | 0.59 | 0.39 | 0.48 | 0.26 | 0.14 | 0.26 | 0.16 | 0.07 | 0.57 | 0.15 | 0.64 | 0.47 | 0.41 |  |  |  |
| **22** | 0.48 | 0.33 | 0.30 | 0.20 | 0.62 | -0.05 | 0.18 | 0.22 | 0.47 | 0.35 | 0.37 | 0.25 | 0.09 | 0.21 | 0.12 | 0.05 | 0.68 | 0.13 | 0.72 | 0.39 | 0.37 | 0.76 |  |  |
| **23** | 0.59 | 0.21 | 0.20 | 0.12 | 0.38 | -0.06 | 0.13 | 0.14 | 0.34 | 0.21 | 0.26 | 0.16 | 0.07 | 0.15 | 0.10 | 0.04 | 0.70 | 0.08 | 0.76 | 0.28 | 0.24 | 0.63 | 0.70 |  |

**Table S4a.** Concordance correlation coefficients for log potencies relative to 16/284 (human panel samples only); values ≥0.8 shaded

| **Lab** | **01** | **02** | **03** | **04** | **05** | **07** | **08** | **09** | **10** | **11a** | **11b** | **12** | **13** | **14a** | **14b** | **15** | **16** | **17** | **18** | **19** | **20** | **21** | **22** | **23** |
| --- | --- | --- | --- | --- | --- | --- | --- | --- | --- | --- | --- | --- | --- | --- | --- | --- | --- | --- | --- | --- | --- | --- | --- | --- |
| **01** |  |  |  |  |  |  |  |  |  |  |  |  |  |  |  |  |  |  |  |  |  |  |  |  |
| **02** | 0.78 |  |  |  |  |  |  |  |  |  |  |  |  |  |  |  |  |  |  |  |  |  |  |  |
| **03** | 0.89 | 0.83 |  |  |  |  |  |  |  |  |  |  |  |  |  |  |  |  |  |  |  |  |  |  |
| **04** | 0.73 | 0.69 | 0.89 |  |  |  |  |  |  |  |  |  |  |  |  |  |  |  |  |  |  |  |  |  |
| **05** | 0.88 | 0.87 | 0.96 | 0.88 |  |  |  |  |  |  |  |  |  |  |  |  |  |  |  |  |  |  |  |  |
| **07** | 0.81 | 0.75 | 0.90 | 0.81 | 0.85 |  |  |  |  |  |  |  |  |  |  |  |  |  |  |  |  |  |  |  |
| **08** | 0.92 | 0.89 | 0.96 | 0.82 | 0.96 | 0.88 |  |  |  |  |  |  |  |  |  |  |  |  |  |  |  |  |  |  |
| **09** | 0.78 | 0.80 | 0.95 | 0.87 | 0.90 | 0.87 | 0.90 |  |  |  |  |  |  |  |  |  |  |  |  |  |  |  |  |  |
| **10** | 0.78 | 0.74 | 0.93 | 0.97 | 0.92 | 0.86 | 0.87 | 0.88 |  |  |  |  |  |  |  |  |  |  |  |  |  |  |  |  |
| **11a** | 0.71 | 0.68 | 0.86 | 0.89 | 0.84 | 0.93 | 0.82 | 0.90 | 0.88 |  |  |  |  |  |  |  |  |  |  |  |  |  |  |  |
| **11b** | 0.80 | 0.72 | 0.93 | 0.94 | 0.94 | 0.87 | 0.89 | 0.91 | 0.94 | 0.94 |  |  |  |  |  |  |  |  |  |  |  |  |  |  |
| **12** | 0.67 | 0.67 | 0.71 | 0.57 | 0.71 | 0.78 | 0.76 | 0.67 | 0.64 | 0.70 | 0.67 |  |  |  |  |  |  |  |  |  |  |  |  |  |
| **13** | 0.49 | 0.51 | 0.69 | 0.82 | 0.64 | 0.66 | 0.59 | 0.70 | 0.83 | 0.72 | 0.71 | 0.44 |  |  |  |  |  |  |  |  |  |  |  |  |
| **14a** | 0.95 | 0.83 | 0.90 | 0.74 | 0.88 | 0.89 | 0.94 | 0.83 | 0.80 | 0.79 | 0.82 | 0.76 | 0.56 |  |  |  |  |  |  |  |  |  |  |  |
| **14b** | 0.46 | 0.58 | 0.52 | 0.53 | 0.60 | 0.57 | 0.63 | 0.51 | 0.54 | 0.58 | 0.57 | 0.73 | 0.38 | 0.58 |  |  |  |  |  |  |  |  |  |  |
| **15** | 0.37 | 0.33 | 0.46 | 0.57 | 0.45 | 0.49 | 0.41 | 0.42 | 0.59 | 0.50 | 0.51 | 0.26 | 0.72 | 0.40 | 0.24 |  |  |  |  |  |  |  |  |  |
| **16** | 0.83 | 0.85 | 0.96 | 0.88 | 0.97 | 0.87 | 0.96 | 0.94 | 0.92 | 0.87 | 0.94 | 0.78 | 0.67 | 0.87 | 0.67 | 0.42 |  |  |  |  |  |  |  |  |
| **17** | 0.65 | 0.66 | 0.83 | 0.87 | 0.80 | 0.70 | 0.73 | 0.80 | 0.89 | 0.73 | 0.81 | 0.41 | 0.84 | 0.66 | 0.33 | 0.66 | 0.77 |  |  |  |  |  |  |  |
| **18** | 0.91 | 0.78 | 0.96 | 0.89 | 0.92 | 0.89 | 0.91 | 0.88 | 0.93 | 0.84 | 0.90 | 0.65 | 0.72 | 0.92 | 0.50 | 0.50 | 0.91 | 0.84 |  |  |  |  |  |  |
| **19** | 0.87 | 0.83 | 0.75 | 0.58 | 0.78 | 0.64 | 0.80 | 0.64 | 0.64 | 0.53 | 0.62 | 0.54 | 0.40 | 0.82 | 0.34 | 0.30 | 0.69 | 0.58 | 0.75 |  |  |  |  |  |
| **20** | 0.70 | 0.83 | 0.65 | 0.49 | 0.67 | 0.59 | 0.72 | 0.61 | 0.54 | 0.49 | 0.52 | 0.67 | 0.34 | 0.70 | 0.41 | 0.20 | 0.65 | 0.43 | 0.60 | 0.80 |  |  |  |  |
| **21** | 0.78 | 0.76 | 0.79 | 0.75 | 0.90 | 0.59 | 0.82 | 0.72 | 0.76 | 0.62 | 0.80 | 0.51 | 0.46 | 0.69 | 0.44 | 0.34 | 0.81 | 0.67 | 0.74 | 0.74 | 0.62 |  |  |  |
| **22** | 0.74 | 0.61 | 0.81 | 0.83 | 0.79 | 0.70 | 0.76 | 0.85 | 0.80 | 0.82 | 0.88 | 0.52 | 0.66 | 0.75 | 0.46 | 0.42 | 0.80 | 0.72 | 0.81 | 0.55 | 0.43 | 0.69 |  |  |
| **23** | 0.44 | 0.54 | 0.63 | 0.72 | 0.68 | 0.74 | 0.60 | 0.66 | 0.73 | 0.81 | 0.74 | 0.54 | 0.61 | 0.52 | 0.51 | 0.43 | 0.69 | 0.60 | 0.62 | 0.34 | 0.36 | 0.50 | 0.44 |  |

**Table S4b.** Concordance correlation coefficients for log potencies relative to 16/322 (human panel samples only); values ≥0.8 shaded

| **Lab** | **01** | **02** | **03** | **04** | **05** | **07** | **08** | **09** | **10** | **11a** | **11b** | **12** | **13** | **14a** | **14b** | **15** | **16** | **17** | **18** | **19** | **20** | **21** | **22** | **23** |
| --- | --- | --- | --- | --- | --- | --- | --- | --- | --- | --- | --- | --- | --- | --- | --- | --- | --- | --- | --- | --- | --- | --- | --- | --- |
| **01** |  |  |  |  |  |  |  |  |  |  |  |  |  |  |  |  |  |  |  |  |  |  |  |  |
| **02** | 0.64 |  |  |  |  |  |  |  |  |  |  |  |  |  |  |  |  |  |  |  |  |  |  |  |
| **03** | 0.92 | 0.73 |  |  |  |  |  |  |  |  |  |  |  |  |  |  |  |  |  |  |  |  |  |  |
| **04** | 0.83 | 0.61 | 0.91 |  |  |  |  |  |  |  |  |  |  |  |  |  |  |  |  |  |  |  |  |  |
| **05** | 0.89 | 0.71 | 0.96 | 0.94 |  |  |  |  |  |  |  |  |  |  |  |  |  |  |  |  |  |  |  |  |
| **07** | 0.80 | 0.76 | 0.86 | 0.72 | 0.81 |  |  |  |  |  |  |  |  |  |  |  |  |  |  |  |  |  |  |  |
| **08** | 0.92 | 0.70 | 0.97 | 0.93 | 0.97 | 0.84 |  |  |  |  |  |  |  |  |  |  |  |  |  |  |  |  |  |  |
| **09** | 0.72 | 0.85 | 0.84 | 0.69 | 0.78 | 0.85 | 0.79 |  |  |  |  |  |  |  |  |  |  |  |  |  |  |  |  |  |
| **10** | 0.84 | 0.60 | 0.91 | 0.96 | 0.95 | 0.73 | 0.94 | 0.66 |  |  |  |  |  |  |  |  |  |  |  |  |  |  |  |  |
| **11a** | 0.74 | 0.72 | 0.83 | 0.76 | 0.81 | 0.93 | 0.82 | 0.88 | 0.71 |  |  |  |  |  |  |  |  |  |  |  |  |  |  |  |
| **11b** | 0.86 | 0.62 | 0.93 | 0.95 | 0.96 | 0.80 | 0.94 | 0.75 | 0.92 | 0.85 |  |  |  |  |  |  |  |  |  |  |  |  |  |  |
| **12** | 0.60 | 0.37 | 0.65 | 0.68 | 0.66 | 0.60 | 0.69 | 0.43 | 0.77 | 0.54 | 0.68 |  |  |  |  |  |  |  |  |  |  |  |  |  |
| **13** | 0.70 | 0.61 | 0.87 | 0.91 | 0.85 | 0.74 | 0.85 | 0.74 | 0.89 | 0.79 | 0.87 | 0.66 |  |  |  |  |  |  |  |  |  |  |  |  |
| **14a** | 0.92 | 0.77 | 0.90 | 0.76 | 0.85 | 0.92 | 0.89 | 0.84 | 0.77 | 0.85 | 0.82 | 0.58 | 0.74 |  |  |  |  |  |  |  |  |  |  |  |
| **14b** | 0.41 | 0.30 | 0.44 | 0.57 | 0.51 | 0.39 | 0.54 | 0.29 | 0.61 | 0.38 | 0.51 | 0.76 | 0.51 | 0.41 |  |  |  |  |  |  |  |  |  |  |
| **15** | 0.71 | 0.49 | 0.78 | 0.87 | 0.82 | 0.72 | 0.81 | 0.59 | 0.83 | 0.75 | 0.88 | 0.56 | 0.82 | 0.69 | 0.47 |  |  |  |  |  |  |  |  |  |
| **16** | 0.84 | 0.64 | 0.95 | 0.96 | 0.96 | 0.77 | 0.96 | 0.73 | 0.97 | 0.77 | 0.95 | 0.77 | 0.91 | 0.81 | 0.61 | 0.81 |  |  |  |  |  |  |  |  |
| **17** | 0.73 | 0.88 | 0.85 | 0.73 | 0.83 | 0.75 | 0.79 | 0.89 | 0.71 | 0.75 | 0.75 | 0.37 | 0.73 | 0.79 | 0.26 | 0.65 | 0.74 |  |  |  |  |  |  |  |
| **18** | 0.95 | 0.74 | 0.97 | 0.87 | 0.93 | 0.86 | 0.94 | 0.81 | 0.88 | 0.82 | 0.89 | 0.61 | 0.83 | 0.95 | 0.42 | 0.75 | 0.89 | 0.84 |  |  |  |  |  |  |
| **19** | 0.87 | 0.82 | 0.83 | 0.68 | 0.81 | 0.76 | 0.81 | 0.76 | 0.71 | 0.67 | 0.71 | 0.44 | 0.59 | 0.88 | 0.29 | 0.56 | 0.70 | 0.84 | 0.87 |  |  |  |  |  |
| **20** | 0.81 | 0.79 | 0.87 | 0.78 | 0.83 | 0.83 | 0.84 | 0.84 | 0.76 | 0.80 | 0.78 | 0.59 | 0.74 | 0.83 | 0.38 | 0.54 | 0.80 | 0.76 | 0.84 | 0.81 |  |  |  |  |
| **21** | 0.76 | 0.53 | 0.78 | 0.85 | 0.89 | 0.53 | 0.81 | 0.56 | 0.86 | 0.56 | 0.84 | 0.52 | 0.65 | 0.61 | 0.45 | 0.68 | 0.82 | 0.66 | 0.73 | 0.69 | 0.66 |  |  |  |
| **22** | 0.78 | 0.61 | 0.80 | 0.75 | 0.77 | 0.68 | 0.77 | 0.80 | 0.68 | 0.80 | 0.82 | 0.44 | 0.76 | 0.80 | 0.33 | 0.67 | 0.73 | 0.74 | 0.79 | 0.69 | 0.70 | 0.63 |  |  |
| **23** | 0.50 | 0.54 | 0.65 | 0.68 | 0.72 | 0.72 | 0.67 | 0.58 | 0.67 | 0.77 | 0.74 | 0.55 | 0.68 | 0.57 | 0.44 | 0.66 | 0.71 | 0.55 | 0.62 | 0.43 | 0.62 | 0.55 | 0.41 |  |

**Table S5.** Summary of inter-laboratory GCV values (%); shading indicates level of inter-laboratory variability

| **Type** | **Sample** | **ED50s** | | **Potencies relative to different reference standards** | | | | | | | | | |
| --- | --- | --- | --- | --- | --- | --- | --- | --- | --- | --- | --- | --- | --- |
|  |  | **(All Data)** | **(With Exclusions)** | **Set A (All Data)** | | | | | **Set B (With Exclusions)** | | | | |
|  |  |  |  | **16/284** | **16/322** | **S7** | **S14** | **S19** | **16/284** | **16/322** | **S7** | **S14** | **S19** |
| **Human** | 1 | 155 | 133 | 42 | 41 | 57 | 55 | 55 | 22 | 31 | 21 | 28 | 29 |
|  | 3 | 156 | 135 | 39 | 31 | 39 | 49 | 26 | 21 | 31 | 12 | 29 | 31 |
|  | 9 | 114 | 103 | 40 | 33 | 24 | 33 | 38 | 18 | 26 | 16 | 34 | 32 |
|  | 11 | 125 | 111 |  | 43 | 25 | 25 | 34 |  | 18 | 7 | 19 | 20 |
|  | 16 | 158 | 113 | 52 | 53 | 34 | 30 | 63 | 23 | 30 | 20 | 29 | 31 |
|  | 18 | 126 | 98 | 38 | 42 | 33 | 38 | 44 | 18 | 18 | 15 | 19 | 23 |
|  | 24 | 157 | 120 | 43 |  | 38 | 41 | 30 | 18 |  | 24 | 20 | 25 |
|  | 26 | 177 | 126 | 52 | 44 | 45 | 48 | 40 | 36 | 41 | 40 | 39 | 45 |
|  | 29 | 143 | 115 | 54 | 37 | 31 | 41 | 22 | 15 | 27 | 16 | 24 | 25 |
|  | 31 | 148 | 162 | 48 | 55 | 28 | 37 | 35 | 40 | 37 | 33 | 35 | 34 |
|  | 37 | 135 | 100 | 68 | 58 | 54 | 72 | 63 | 32 | 34 | 28 | 31 | 30 |
|  | 38 | 166 | 105 | 108 | 89 | 96 | 112 | 116 | 39 | 31 | 37 | 24 | 29 |
| **Calibration** | 2 | 138 | 70 | 48 | 54 | 39 | 39 | 65 | 33 | 28 | 25 | 18 | 27 |
|  | 7 | 98 | 54 | 27 | 37 |  | 22 | 36 | 21 | 28 |  | 19 | 20 |
|  | 14 | 136 | 59 | 31 | 55 | 22 |  | 41 | 17 | 16 | 19 |  | 10 |
|  | 19 | 116 | 62 | 33 | 29 | 36 | 41 |  | 19 | 23 | 20 | 10 |  |
|  | 36 | 172 | 67 | 63 | 47 | 55 | 72 | 55 | 29 | 29 | 26 | 29 | 26 |
| **Paediatric** | 5 | 178 | 84 | 68 | 63 | 77 | 81 | 94 | 48 | 44 | 68 | 60 | 60 |
|  | 8 | 159 | 117 | 59 | 37 | 50 | 66 | 57 | 33 | 30 | 28 | 28 | 33 |
|  | 23 | 182 | 119 | 75 | 56 | 79 | 87 | 94 | 41 | 29 | 52 | 39 | 45 |
|  | 33 | 169 | 159 | 63 | 64 | 54 | 71 | 54 | 30 | 31 | 23 | 20 | 20 |
| **Vaccinee** | 4 | 129 | 65 | 59 | 55 | 46 | 64 | 55 | 25 | 24 | 22 | 29 | 34 |
|  | 6 | 179 | 166 | 71 | 70 | 44 | 57 | 59 | 35 | 35 | 32 | 37 | 41 |
|  | 12 | 142 | 146 | 53 | 62 | 48 | 56 | 49 | 35 | 42 | 33 | 28 | 31 |
|  | 15 | 111 | 100 | 39 | 57 | 17 | 28 | 36 | 26 | 32 | 15 | 23 | 26 |
|  | 17 | 129 | 95 | 63 | 69 | 64 | 73 | 64 | 59 | 56 | 73 | 69 | 63 |
|  | 20 | 127 | 110 | 67 | 57 | 51 | 63 | 49 | 38 | 36 | 36 | 30 | 34 |
|  | 21 | 125 | 55 | 71 | 76 | 28 | 41 | 49 | 72 | 70 | 22 | 32 | 30 |
|  | 25 | 138 | 89 | 72 | 68 | 63 | 74 | 63 | 35 | 40 | 35 | 39 | 42 |
|  | 28 | 186 | 124 | 72 | 52 | 73 | 80 | 66 | 37 | 36 | 26 | 26 | 35 |
|  | 30 | 139 | 122 | 47 | 45 | 44 | 55 | 35 | 22 | 30 | 27 | 28 | 31 |
|  | 34 | 165 | 173 | 94 | 92 | 94 | 119 | 92 | 32 | 35 | 37 | 35 | 42 |
|  | 35 | 139 | 136 | 52 | 53 | 40 | 58 | 47 | 28 | 32 | 31 | 26 | 23 |
| **Animal** | 13 | 240 | 156 | 131 | 107 | 141 | 124 | 153 | 83 | 63 | 121 | 95 | 98 |
|  | 27 | 227 | 162 | 110 | 76 | 142 | 125 | 126 | 52 | 44 | 74 | 63 | 71 |
| **mAb** | 10 | 164 | 151 | 90 | 103 | 91 | 80 | 98 | 80 | 95 | 70 | 68 | 60 |
|  | 22 | 129 | 113 | 81 | 97 | 82 | 79 | 85 | 61 | 80 | 50 | 59 | 50 |

Shading indicates level of inter-laboratory variability: darker red = increased variability

Blue shading: animal and mAb samples are shaded in blue as they behave differently to human serum samples

Grey shading: empty cell

**Table S6.** Thermal degradation assessment of 16/284; anti-RSV neutralization titres after 6 months storage

| Storage Temperature (°C) | 1 | 2 | GM | % of -20°C |
| --- | --- | --- | --- | --- |
| 4 | 1603 | 1750 | 1675 | 112 |
| 20 | 1263 | 1439 | 1348 | 91 |
| 37 | 1069 | 865 | 962 | 65 |
| 45 | 727 | 710 | 718 | 49 |
| 56 | 81 | 76 | 78 | 5 |

**Table S7.** Thermal degradation assessment of 16/322; anti-RSV neutralization titres after 6 months storage

| Storage Temperature (°C) | 1 | 2 | GM | % of -20°C |
| --- | --- | --- | --- | --- |
| 4 | 791 | 994 | 887 | 126 |
| 20 | 641 | 981 | 793 | 112 |
| 37 | 607 | 619 | 613 | 87 |
| 45 | 610 | 561 | 585 | 83 |
| 56 | 13 | 13 | 13 | 2 |

**Table S8.** Thermal degradation assessment of reconstituted 16/284; percentage relative to 16/284 stored at -20°C and reconstituted on day of assay

| Storage Temperature (°C) | 1 week | 2 weeks | 4 weeks |
| --- | --- | --- | --- |
| 4 | 166 |  | 76 |
| 22 | 105 |  | 123 |
| 37 | 93 | 84 |  |

**Table S9.** Thermal degradation assessment of reconstituted 16/322; percentage relative to 16/322 stored at -20°C and reconstituted on day of assay

| Storage Temperature (°C) | 1 week | 2 weeks | 4 weeks |
| --- | --- | --- | --- |
| 4 | 88 |  | 89 |
| 22 | 89 |  | 113 |
| 37 | 59 | 54 |  |
